# Metabolome and Transcriptome Analysis Reveals Putative Genes Involved in Anthocyanin Accumulation and Coloration in White and Pink Tea (*Camellia sinensis*) Flower

**DOI:** 10.3390/molecules25010190

**Published:** 2020-01-02

**Authors:** Caibi Zhou, Xin Mei, Dylan O’Neill Rothenberg, Zaibo Yang, Wenting Zhang, Shihua Wan, Haijun Yang, Lingyun Zhang

**Affiliations:** 1 Department of Tea Science, Qiannan Normal University for Nationalities, Duyun 558000, China; teasky@foxmail.com (C.Z.); yzb1976110@sohu.com (Z.Y.); 2 College of Horticulture, South China Agricultural University, Guangzhou 510640, China; Dylan.Rothenberg@colorado.edu (D.O.R.); wendyzhang998@163.com (W.Z.); 15797708411@163.com (S.W.); hjyang@scau.edu.cn (H.Y.); 3 South China Botanical Garden, Chinese Academy of Sciences, Guangzhou 510650, China; xmei@scbg.ac.cn

**Keywords:** *Camellia sinensis* (L.), metabolite profiling, RNA-seq, flower color, anthocyanin biosynthesis

## Abstract

A variant of tea tree (*Camellia sinensis* (L.)) with purple buds and leaves and pink flowers can be used as a unique ornamental plant. However, the mechanism of flower coloration remains unclear. To elucidate the molecular mechanism of coloration, as well as anthocyanin accumulation in white and pink tea flowers, metabolite profiling and transcriptome sequencing was analyzed in various tea flower developmental stages. Results of metabolomics analysis revealed that three specific anthocyanin substances could be identified, i.e., cyanidin *O*-syringic acid, petunidin 3-*O*-glucoside, and pelargonidin 3-*O*-β-d-glucoside, which only accumulated in pink tea flowers, and were not able to be detected in white flowers. RNA-seq and weighted gene co-expression network analysis revealed eight highly expressed structural genes involved in anthocyanin biosynthetic pathway, and particularly, different expression patterns of flavonol synthase and dihydroflavonol-4-reductase genes were observed. We deduced that the disequilibrium of expression levels in flavonol synthases and dihydroflavonol-4-reductases resulted in different levels of anthocyanin accumulation and coloration in white and pink tea flowers. Results of qRT-PCR performed for 9 key genes suggested that the expression profiles of differentially expressed genes were generally consistent with the results of high-throughput sequencing. These findings provide insight into anthocyanin accumulation and coloration mechanisms during tea flower development, which will contribute to the breeding of pink-flowered and anthocyanin-rich tea cultivars.

## 1. Introduction

Tea (*Camellia sinensis* (L.) O. Kuntze) is the most popular beverage in the world, largely because of its beneficial health attributes [1,2]. Additionally, it is recognized as one of the most central economic crops in China, India and other developing countries, functioning as a powerful driver of poverty reduction in poor mountainous regions and underdeveloped rural economies. Although much research has been carried out on primary metabolic processes in different varieties of tea during bud and leaf development [3,4,5,6], few studies have focused on the metabolism of effective components during tea flower development. The reason may be that almost all tea plants have white flowers that typically bloom at the end of harvesting season, and these flowers are rarely used due to the low content of healthful compounds. However, some studies showed that total content of catechins and flavonoids in tea flowers were similar to that of leaf, but contained less caffeine [7]. Furthermore, the extract of tea flowers has demonstrated antioxidant properties, due to strong free radical scavenging abilities [8]. Therefore, it is interesting from the perspectives of natural flavor and natural healthful food to study the accumulation of these beneficial components during flower development.

For ornamental plants, an important feature is that flower color is closely related to pigment distribution type [9]. Numerous studies showed that carotenoids, flavonoids, and alkaloids are among the most principal pigments in flower color formation [10,11,12]. Particularly, carotenoids and flavonoids have attracted more attention because they are widely distributed pigments in plants that produce brilliant colors in petals. Carotenoids can demonstrate various colors from brilliant red, orange to yellow in flowers [13]. While flavonoids, as the most important secondary metabolites in plants, can produce pale yellow to purple colors, depending on flavonoids type. As an ornamental plant, the chemical composition, as well as coloration in *Camellia* or *Camellia (Theaceae)* flowers has been widely researched. Zhou et al. [14] found that the principal pigments in the golden yellow flowers (*Camellia nitidissima* Chi.) are carotenoids and flavonol glycosides. Jianbin Li et al. [15] analyzed the pigment composition of red-flowered *Camellia* species, and found that cyanidin-core structure pigments (such as cyanidin 3,5-*di*-*O*-β-glucoside) can yield the most dominant phenotype among wild red-flowered Camellia species in China. Norihiko et al. [16] found the anthocyanins were also contained in different compositions in the red flowers of Benibana-cha.

Despite some research indicating that anthocyanins are absent in many white and yellow flowers of ornamental plants [17], almost all core metabolites involved in anthocyanin synthesis were detected in the white grape hyacinth petal [18]. Similarly, the highest content of proanthocyanidins was detected in sepals between the stages of complete separation and full bloom in white tea flower (*Camellia sinensis*) development [19].

In recent years, certain anthocyanin-rich purple tea varities, such as Zijuan (*Camellia sinensis* var. *assamica*) and Zixin, have been extensively studied for their anthocyanin accumulation mechanisms during leaf development [4,6,20,21,22]. However, mostly all of these purple-leaf *Camellia sinensis* plants have white flowers, including Zijuan and Zixin. On a chance discovery, our research team found a natural mutant, an anthocyanin-rich purple bud tea with pink flowers. As a rather unique variety of tea tree, the mutant can provide insight into different mechanisms of coloration, and offer an opportunity to develop a more comprehensive understanding of anthocyanin accumulation in white and pink tea flowers.

In the current study, our primary goal was to elucidate anthocyanin metabolism and purple coloration in the flowers of *C. sinensis*. Our means to attain this goal included first the classification of the primary differential metabolites during white and pink tea flower development using metabolomics analysis. Next key structural genes involved in the anthocyanin biosynthetic pathway during different stages of tea flower development were obtained through analysis of differentially expressed genes (DEGs) and KEGG enrichment analysis. This study can provide a systems-level context for further studies on the gene regulation network of anthocyanin biosynthesis pathways involved in flower color formation in ornamental camellias, and contribute to the breeding of pink-flowered tea cultivars.

## 2. Results

### 2.1. Anthocyanin Content in White and Pink Tea Flower

**Figure 1:**
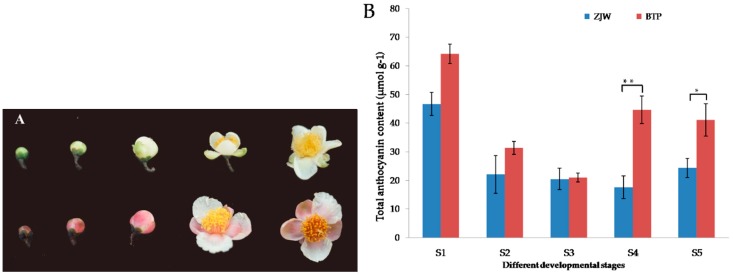
Color phenotypes and anthocyanin contents of white (ZJW) and pink (BTP) flowers during 5 developmental stages (S1–S5). (**A**) The phenotypes of five development stage of ZJW (white) flower and BTP (pink). (**B**) Anthocyanin content (µmol/g fresh weight) of white flower (ZJW) and pink flower (BTP) samples. S1, mature flower bud; S2, pre-opening; S3, initial opening; S4, half bloom; S5, full bloom. Each data is the mean of three biological replicates. * *p* < 0.01; ** *p* < 0.05.

To evaluate biochemical differences in white and pink tea flower development stages, the anthocyanin content and metabolic profiles of tea petals were compared. The appearance of the pink tea flower discovered by our research team, referred to as BTP, showed more pink pigments than white tea flower, referred to as ZJW (Figure 1B). The anthocyanin extracted from tea petals was confirmed to be anthocyanin in subsequent HPLC-MS and multiple reaction monitoring (MRM) analysis (Table 1). The results show that the pink tea flower contained higher levels of anthocyanin and more of anthocyanin accumulation than the white tea flower. BTP S1 contained higher levels of anthocyanin compared to other developmental stages of the pink tea flower. A low but measurable content of anthocyanin components was observed in the white tea flower, which may be due in part to its anthocyanin-rich genetic background, however the content of total anthocyanins in white flowers was much less than in pink flowers (Figure 1A). Particularly, the content of colored anthocyanin glycosides was lower in white flower than pink flower (Table 1).

### 2.2. Metabolomics Analysis of Tea Flower Development Stages

**Figure 2:**
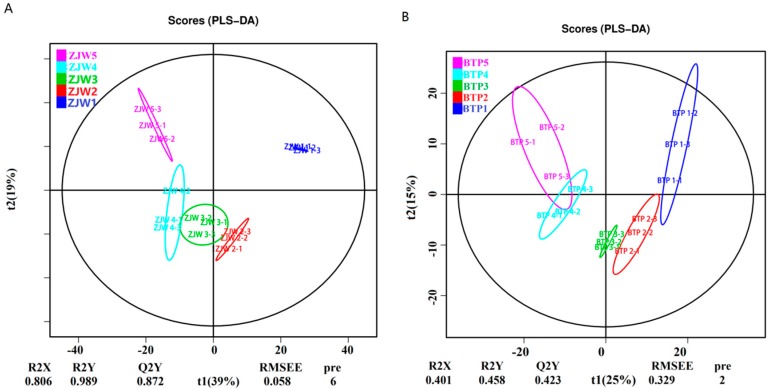
PLS-DA score plots generated from PLS-DA models in different development stages of tea flower. (**A**) PLS-DA score plot of ZJW flower; R2X = 0.806, R2Y = 0.989, Q2Y = 0.872; (**B**) PLS-DA score plot of BTP flower; R2X = 0.401, R2Y = 0.458, Q2Y = 0.243; RMSEE represent Root Mean Square Error of Estimation.

Partial least squares-discriminant analysis (PLS-DA) is a useful and reliable algorithm that can be used for discriminative variable selection as well as for predictive and descriptive modeling. The advantage of this method is that it can clarify the distinction between groups of observations, helping to explain which variables transmit the class-defining information. To compare the metabolites involved in the pigmentation of the two different tea flowers, score plots of PLS-DA analysis of the supervised model was performed. The results showed that the metabolic profiles in three different development stages were significantly changed (Figure 2). As shown in Figure 2A, the scores plot of PLS-DA model discriminated the white tea flower development stages, with different samples all exhibiting satisfactory separation, as reflected by all stages having significantly different metabolite compositions. In Figure 2B, the scores plot of PLS-DA modeling show a different clustering tendency. The figure shows that the metabolites of the first stage were similar to the second and third stages, and the metabolites of fourth stage were similar to fifth stage. These results indicate that the metabolic profiles were strikingly different between ZJW flower development stages, while the metabolic profiles were similar between BTP flower development stages.

**Figure 3:**
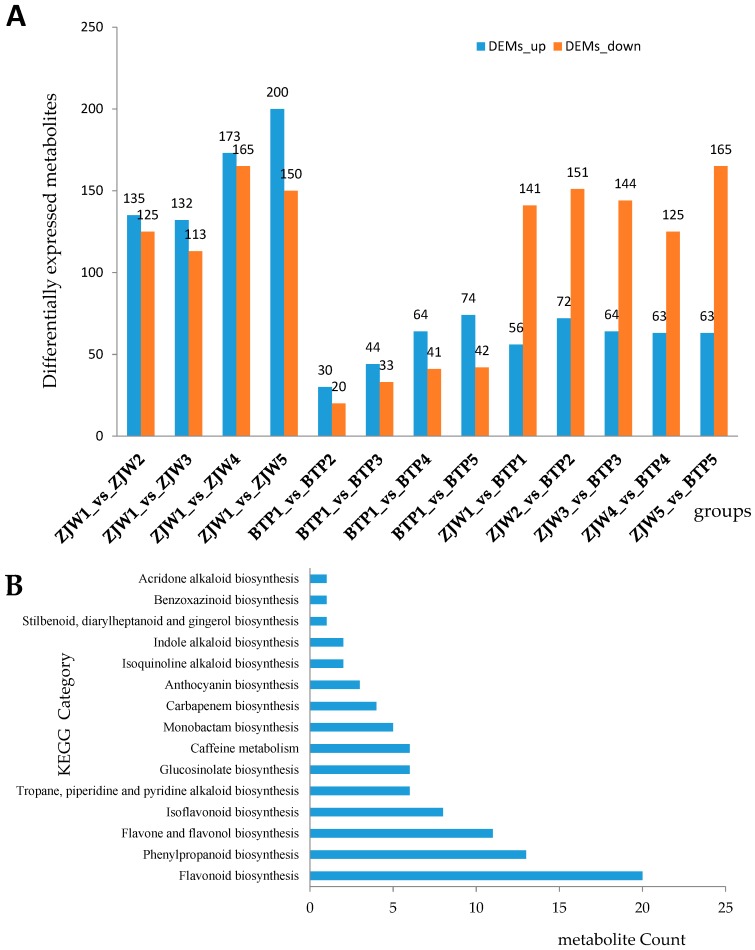
The number of differentially expressed metabolites (DEMs) and the number of KEGG pathway annotations for secondary metabolism. (**A**) DEMs in ZJW and BTP tea flower. (**B**) The number of KEGG pathway annotations involved in secondary metabolism of ZJW and BTP tea flower from S1 to S5.

In this paper, 638 metabolites were identified by metabolomics analysis. All tea flower metabolites at different developmental stages (from S1 to S5) were studied to detect differentially expressed metabolites (DEMs) between ZJW and BTP. During white tea flower development, the number of DEMs in ZJW1 vs. ZJW2, ZJW1 vs. ZJW3, ZJW1 vs. ZJW4 and ZJW1 vs. ZJW5 showed an increasing trend. In pink tea flowers, the BTP1 vs. BTP2, BTP1 vs. BTP3, BTP1 vs. BTP4 and BTP1 vs. BTP5 showed a trend similar to ZJW (Figure 3A). Comparing ZJW and BTP from the first to the fifth stage, the number of metabolites that were downregulated was higher (from 125 to 165) than those that were upregulated (from 56 to 72).

DEMs between ZJW and BTP flowers at different development stages indicated enriched metabolism of other secondary metabolites and amino acids (Figure 3B and Table S1). Through DEM venn analysis (Supplementary Materials Figure S1) and KEGG pathway annotation of metabolites, 52 DEMs of a variety of functional pathways were discovered, among which 13 DEMs were involved in the anthocyanin biosynthetic pathway (Table 1). In particular, cyanidin *O*-syringic acid, petunidin 3-*O*-glucoside, and pelargonidin 3-*O*-beta-d-glucoside were found to be abundant in pink tea flowers, whereas they could not be detected in white tea flowers. The content of cyanidin 3-*O*-glucoside was 10 times higher in pink flowers than in white flowers (Table 1). The results indicated that these anthocyanins might be playing a crucial role in the color formation of BTP flowers.

### 2.3. RNA Sequencing and Sample Transcriptome Mapping

All development stages of the tea petal samples were subject to RNA-seq analysis in order to discover possible molecular mechanisms of color formation in white and pink tea flowers. After raw read quality filtering, 396,898,121 and 345,827,515 clean reads were obtained from ZJW and BTP libraries respectively. The clean reads of each sample were greater than 6.22 GB, Q30 percentage was over 85.01% and the average content of GC was 44.79% (Table S2). The *Camellia sinensis var. assamica* genome database was used as a reference genome onto which 72.37–78.46% of the clean reads were mapped. Among the mapped reads, the uniquely mapped reads accounted for 70.03–75.98% (Table S3). Overall, these data implied that the sample RNA sequencing was of reliable quality and could be used for further investigation.

### 2.4. Differentially Expressed Genes (DEGs) in BTP and ZJW Flower Development

**Figure 4:**
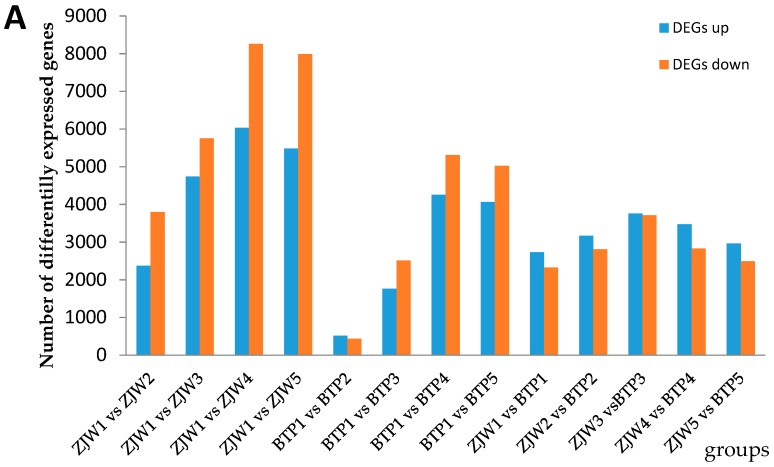

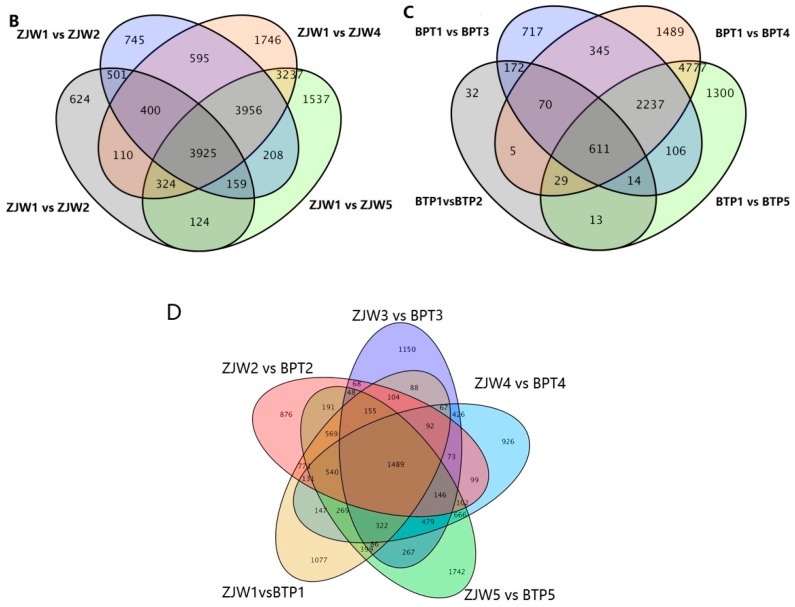
DEGs statistics and Venn diagrams between different cDNA libraries. (**A**) The number of DEGs between ZJW and BTP developmental stages. (**B**) Venn Diagram of DEGs between white flower tea development stages. (**C**) Venn Diagram of DEGs between pink flower tea development stages. (**D**) Venn diagram of DEGs between ZJW and BTP at the 5 developmental stages.

To identify the DEGs involved in tea flower coloration, the fragment per kilobase of exon per million fragments mapped (FPKM) values were analyzed for each gene in white and pink tea flowers at different developmental stages (base on false discovery rate ≤ 0.01, and fold change ≥ 2). A total of 6167, 10489, 14293 and 13470 DEGs were identified in the pairwise comparisons of ZJW1 vs. ZJW2, ZJW1 vs. ZJW3, ZJW1 vs. ZJW4 and ZJW1 vs. ZJW5, respectively. In BTP flowers, 946, 4272, 9563 and 9087 DEGs were identified in the pairwise comparisons of BTP1 vs. BTP2, BTP1 vs. BTP3, BTP1 vs. BTP4 and BTP1 vs. BTP5 (Figure 4A and Table S4). The amount of down-regulated DEGs was more than that of up-regulated genes during the two flowers’ development (Figure 4A). The number of DEGs in the third stage was the highest compared to the other stages between the two tea flowers, suggesting that the third stage may be a key stage for tea flower color formation, although more research is necessary to confirm this supposition.

3925 DEGs were identified using Venn analysis by comparing ZJW white flowers at different development stages (Figure 4B), 611 DEGs by comparing BTP pink flowers at different development stages (Figure 4C), and 1489 DEGs by comparing ZJW versus BTP (Figure 4D).

**Figure 5:**
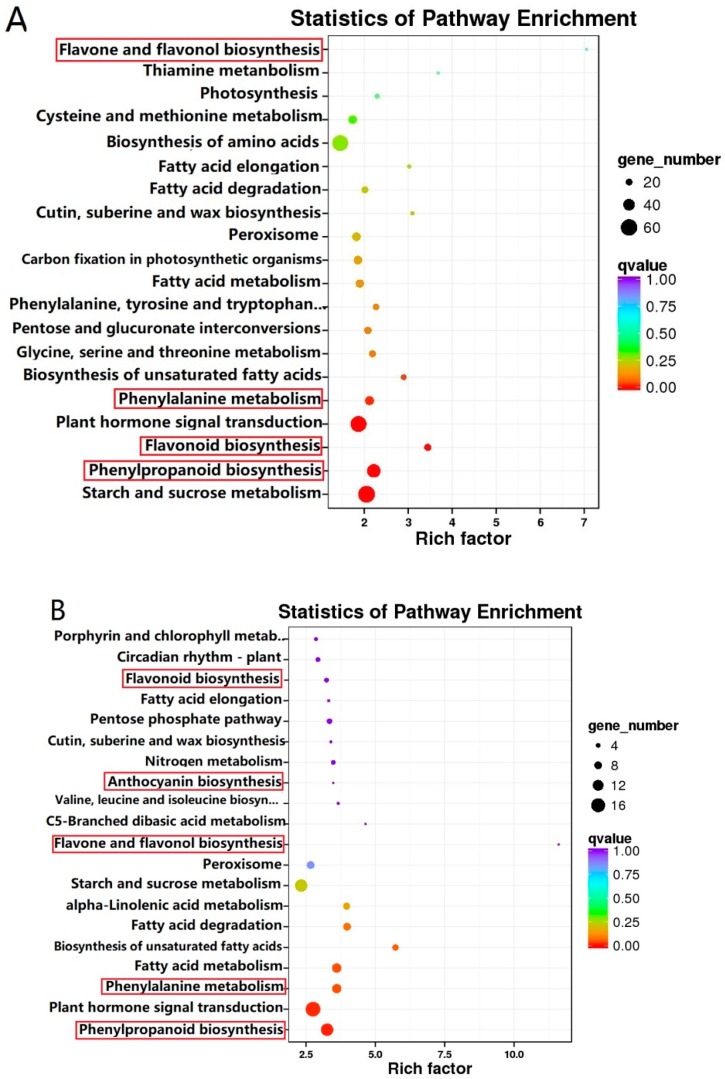
Results of top 20 KEGG enriched pathways in white (**A**) and pink (**B**) tea flower development stages. Q-value is a p-value that has been adjusted for the false discovery rate (FDR). A lower q-value indicates that a lower percentage of significant results will be false positives. Rich factor represents the degree of enrichment (i.e., over-representation) of genes under the designated pathway term. Greater the value of the rich factor, greater is the degree of pathway enrichment.

In order to classify the genes involved in the flavonoid biosynthetic pathway, all DEGs were subject to KEGG pathway enrichment analysis [23]. 115 biosynthesis and metabolism pathways were enriched in the pairwise comparison of ZJW flower development, in which phenylpropanoid biosynthesis (ko00940, 47 genes), phenylalanine metabolism (ko00360, 29 genes), flavonoid biosynthesis (ko00944, 21 genes), flavone and flavonol biosynthesis (ko00944, 3 genes) involved in the flavonoid pathway were identified (Figure 5A and Table S6). The DEGs were enriched to 81 KEGG pathways in the pairwise comparison of BTP flower development. Genes involved in flavonoid biosynthesis included those integral to phenylpropanoid biosynthesis (ko00940, 14 genes), phenylalanine metabolism (ko00360, 10 genes), flavonoid biosynthesis (ko00944, 4 genes), flavonol and flavone biosynthesis (ko00944, 1 gene) and anthocyanin biosynthesis (ko00944, 1 gene) (Figure 5B and Table S7). In the comparison of ZJW versus BTP, 1489 DEGs were most highly enriched in anthocyanin biosynthesis (ko00944, 3 genes), flavonoid biosynthesis (ko00944, 4 genes), all phenylpropanoid biosynthesis (ko00940, 3 genes), all of which are involved in flavonoid biosynthetic pathway.

Based on above KEGG pathway enrichment results, the identified genes involved in phenylalanine and flavonoid metabolic pathways were rigidly annotated using KOG, Swiss.Prot, eggNOG and NR database. Unigenes involved in the flavonoid biosynthesis pathway were identified with the FPKM values > 20. Finally, two UDP-glucosyl transferase genes were identified in both the white and pink flower, including UDP-glucose (glucosyl transferase) and flavonoid 3-*O*-galactosyl transferase associated with anthocyanin biosynthesis in the tea flower’s first development stage. Also, two chalcone synthase 2 (CHS2, CSA029707, CSA026009), flavonoid-3′-hydroxylase (F3′H, CSA023049), one flavanone 3-hydroxylase (F3H, CSA004930), one flavonol synthase (FLS, CSA008358), three leucoanthocyanidin reductase (LAR, CSA019984, CSA018523, CSNG5035), one anthocyanidin reductase (ANR, CSA011986), two dihydroflavonol-4-reductase (DFR, CSA003949, CSA035727) and two flavonoid 3′,5′-methyltransferase (F3′5′H, CSNG 52642, CSNG 39835) were identified in the second developmental stage. Similarly, all highly expressed structural genes involved in the flavonoid pathway were identified in the flower development stages (Table S8).

### 2.5. Co-Expression Analysis of Genes Related to the Flavonoid Biosynthesis Pathway

**Figure 6:**
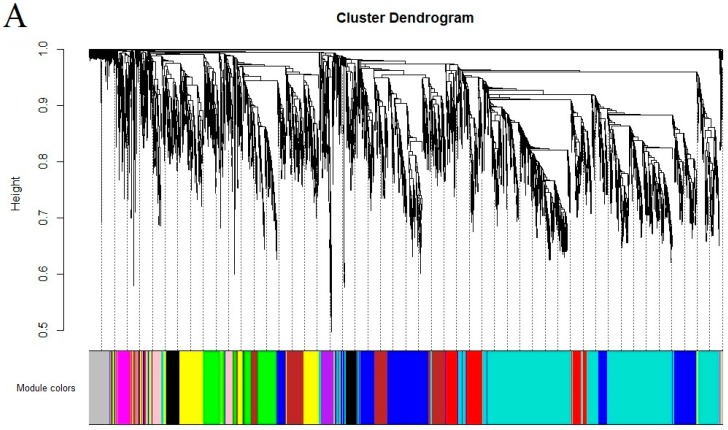

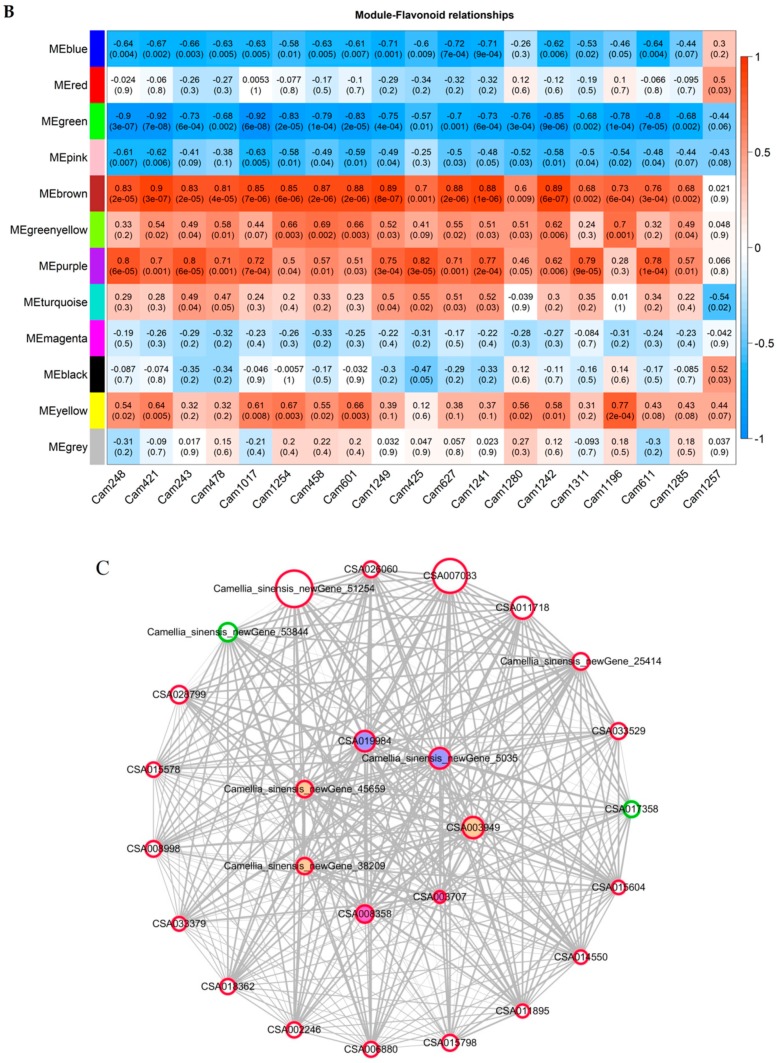
WGCNA of gene expressions and the related traits. (**A**) Hierarchical clustering tree showing co-expression modules. Genes within the same color-coded module represent a group of genes with similar expression patterns. (**B**) Module-trait relationship. The cells in Figure 6A were assigned a color based on their statistical significance and labeled with two numbers; the lower number indicates the p-value and the upper number indicates the correlation coefficient. The size of node circle is positively correlated with the number of the interacting genes. (**C**) Correlation networks of hub genes in the brown and purple module.

In order to examine the regulatory network of genes during tea flower development and color formation, all unigenes were subjected to WGCNA. According to standard WGCNA procedure, color-coded modules were created to represent a group of highly interconnected genes. Results showed that 12 modules were identified by applying the cut tree dynamic function (Figure 6A,B), and brown- and purple-coded modules were significantly correlated with flavonoid components. The correlation coefficients between each module with flavonoid DEMs are shown in Figure 6B. The brown module contained 466 DEGs, and the purple module contained 101 DEGs (Table S9). Network construction highlighted hub genes expected to play crucial roles in flavonoid accumulation using Cytoscape. Hub genes were then discovered based on their eigengene connectivity value (KME) ≥ 0.99 and edge weight value ≥0.5 in the gene network. The hub genes were visualized using Cytoscape software (3.6.1 version) and illustrated in Figure 6C [24].

In the present study, we identified 26 hub genes from the brown and purple modules encoding different proteins, including two FLS genes, three DFR genes, two LAR genes and two GDP-L-galactose phosphorylase (GGP) genes (Table S10). Among the 26 hub genes, purple acid phosphatase (PAP) and ferric reduction oxidase 8 (FRO8) were firstly identified, which are involved in carbohydrate transport and metabolism and inorganic ion transport and metabolism, respectively.

Transcription factors (TFs) are well known to play a crucial role in secondary metabolite biosynthesis in plants. In this study, two special TFs were detected in brown and purple modules by WGCNA, namely, ultraviolet-B receptor (UVR8) and GATA transcription factor 4 (GATA zinc finger, GATA4). Previous studies indicated that UV-B could induce CHS expression and affect flavonoid accumulation [25]. Furthermore, functional annotation detected hub genes involved in biological regulation (GO:0065007) and catalytic activity (GO:0003824), such as cyclin-P3-1 and alkaline and neutral invertase.

### 2.6. Verification of Anthocyanin Biosynthesis-Related Genes

**Figure 7:**
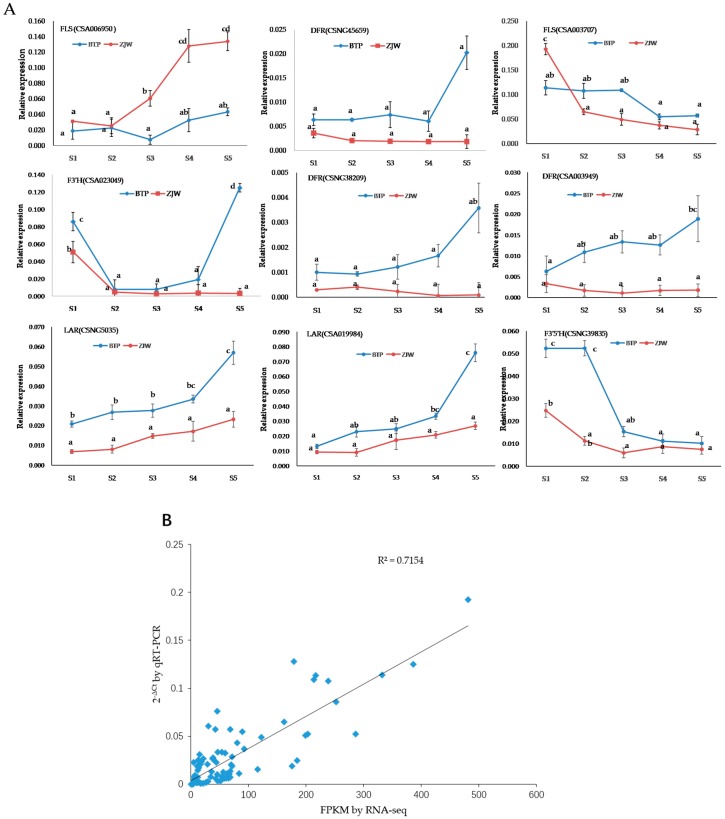
Quantitative real-time PCR (qRT-PCR) validation of 9 structural genes. (**A**) Expression patterns of 9 genes involved in anthocyanin biosynthesis in white and pink tea flower development stage (from S1 to S5). Each column represents an average of three biological replicates, with standard errors indicated by vertical bars. Values with a different accompanying letter are statistically significantly different according to Duncan’s multiple range test at *p* < 0.05. (**B**) Correlation of the expression levels of 9 selected genes measured by qRT-PCR and RNA-seq. S1, mature flower bud; S2, pre-opening; S3, initial opening; S4, half bloom; S5, full bloom.

To confirm the expression profiling of key DEGs involved in the anthocyanin pathway, RNA-seq and the resulting gene expression patterns of the 9 DEGs (i.e., F3′H, FLS, DFR, LAR, F3′5′H) showing the most significantly differential expression during tea flower development were subject to quantitative real-time PCR. The primer sequences for each gene are listed in Table S5. Pearson’s correlation coefficient was used to establish the correlation between qRT-PCR and RNA-seq results of the same development stages. The qRT-PCR and RNA-seq data were highly correlated, and exhibited consistency in the up- and downregulated expression of DEGs (Figure 7B). The correlation coefficient between FPKM value by RNA-seq and qRT-PCR was 0.7155 (Figure 7B). These results indicate that the RNA-seq data were sound and credible.

**Figure 8:**
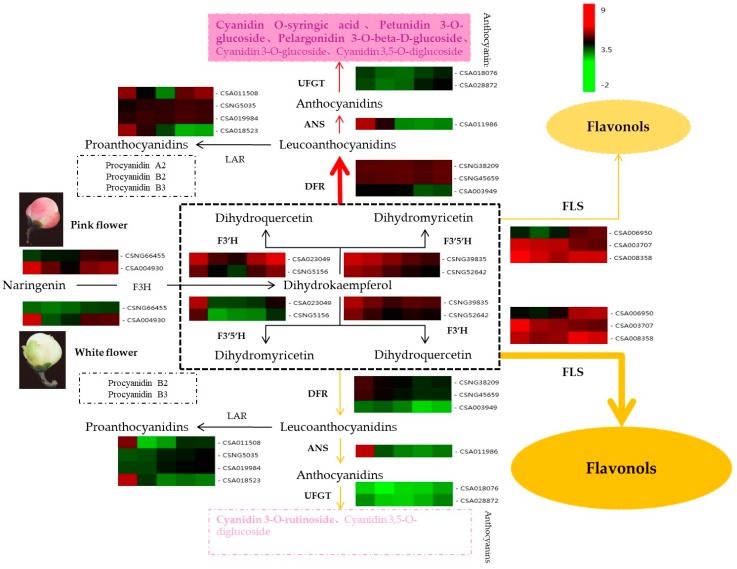
Tea flower unigenes thought to be incorporated in the anthocyanin biosynthetic pathway and their genes expression levels. The heatmap is created according to the average expression levels of related biosynthetic genes based on FPKM value. A color bar is presented at the top right. Green indicates low expression, and red indicates high expression. DFR gene variants were all significantly upregulated in pink flowers relative to white flowers. DFR is responsible for catalyzing the transformation of dihydroquercetin and dihydromyricetin into leucoanthocyanidins, which are upstream precursors to anthocyanins in the anthocyanin biosynthetic pathway. The significant upregulation of three DFR genes indicates that flavonoid metabolism in purple flowers shunts more substrate than white flowers into the anthocyanin biosynthesis pathway. Likewise, qRT-PCR revealed that in white flowers high FLS activity shunted substrate away from the anthocyanin pathway into the flavonol biosynthesis pathway. The relatively high FLS activity may explain the white coloration in ZJW flowers, while the relatively high DFR activity may explain the pink coloration in in BTP flowers. Abbreviations are as follows: DFR (dihydroflavonol reductase); F3H (flavanone-3-hydroxylase); F3′H (flavonoid 3′-hydroxylase); F3′5′H (flavonoid 3′,5′-hydroxylase); FLS (flavonol synthase); LAR (leucoanthocyanidin reductase). The heatmap was prepared using the Cluster program on biocloud.net.

Our findings concerning qRT-PCR were useful in elucidating the mechanisms of white and pink tea flower coloration. Firstly, three DFR gene variants were all significantly upregulated in pink flowers relative to white flowers. DFR is responsible for catalyzing the transformation of dihydroquercetin and dihydromyricetin into leucoanthocyanidins, which are upstream precursors to anthocyanins in the anthocyanin biosynthetic pathway (Figure 8). The significant upregulation of three DFR genes indicates that flavonoid metabolism in purple flowers shunts more substrate than white flowers into the anthocyanin biosynthesis pathway. Likewise, qRT-PCR revealed that in white flowers high FLS activity shunted substrate away from the anthocyanin pathway into the flavonol biosynthesis pathway. The relatively high FLS activity may explain the white coloration in ZJW flowers, while the relatively high DFR activity may explain the pink coloration in in BTP flowers (Figure 7A and Figure 8).

## 3. Discussion

### 3.1. Effect of Content and Types of Anthocyanins on Coloration in White and Pink Tea Flowers

Camellia plants belonging to the tea family (Theaceae) are evergreen shrubs, and some of them are notable as beautiful ornamental flowering species. Of course, a famous subspecies is *Camellia sinensis* (L.), which is the source of tea. The color of common camellia flowers, such as *C. reticulata, C. sasanqua* and *C. mairei,* range from white through pink to red and variegated. However, the flowers of *Camellia sinensis* (L.) have nearly exclusively been observed to be white in color. Exceptionally, two new mutations with red and pink flowers were recently found, namely Benibana-cha [16] and Baitang purple tea (BTP), respectively. In order to reveal the mechanism of color formation in camellia plants, many studies have analyzed the chemical composition of several types of camellia flowers. Multiple studies have confirmed that the color of camellia flowers relates to the content and composition of flavonoids, such as flavonols, flavones and anthocyanins. A similar study in ornamental plants verified cyanidin and peonidin were responsible for red color [26] and flavonols and flavones share important roles in yellow coloration [27]. Virtually without exception, three delphinidin and cyanidin anthocyanins were identified in red flower tea (Benibana-cha) [16], and five cyanidin anthocyanins were identified in *Camellia* (Dalicha) [28,29]. Li et al. [15] have verified that the taxonomic relationships of wild camellias can be identified using 25 delphinidin and cyanidin anthocyanins from different wild camellia flowers. In the development of golden flower tea (*Camellia nitidissima*), anthocyanins were not detected [14].

In this study, flower color difference was found to be related to the amount of total anthocyanins and flavonoids, particularly total anthocyanin content. Total anthocyanin content increased with pink flower development, while minute levels of anthocyanin accumulation were detectable in white flowers, which is concomitant with results obtained from coloration analysis in other pink and white ornamental plants [18,30,31]. Additionally, certain anthocyanin compounds were found to only accumulate in pink tea flowers, particularly cyanidin 3-*O*-glucoside and pelargonin (pelargonidin 3,5-di-*O*-glucoside), which were found in quantities ten times higher than white flowers, implying that the significant variations in anthocyanin accumulation led to different coloration in white and pink tea flowers. This reasoning is in accordance with previous studies on ornamental plants, such as that by Wang et al. [10] which found a relationship between pink flower coloration and the content of pelargonidin 3-*O*-glucoside, cyanidin 3-*O*-glucoside, pelargonidin 3,5-di-*O*-glucoside and peonidin 3-*O*-glucoside in tree peony.

In nature, cyanidin is a reddish-purple pigment, while pelargonidin lends an orange hue to flowers and reddish tones to several fruits and berries [32]. Some studies have shown that blue flowers have delphinidin-based anthocyanidin and red flowers have pelargonidin-based anthocyanidin [30]. The color of pink flower Albert Greenberg and Roxburgh lily can be deduced by the type of delphinidin and cyanidin derivatives, and deeper pink flowers had the maximal amount of cyanidin derivatives. At the same time, no anthocyanins were detected in white and yellow flowers [31].

Li Xue et al. [27] found that cyanidin 3-*O*-glucoside was the primary anthocyanin in pink-flowered strawberry, and pelargonidin and delphinidin derivatives were also found in low levels. Similarly, peonidin 3-*O*-glucoside, cyanidin 3-*O*-glucoside and cyanidin 3,5-*O*-diglucoside were detected in the pink petals of the ornamental tree *Prunus mume,* while no compounds related to these secondary metabolites were detected in white petals [11]. He et al. [33] similarly found that anthocyanins were detectable in purple, red, and orange *Lycoris longituda*, however no anthocyanins were found in white and yellow flowers. Recent results indicate the white-flowered *Nicotiana sylvestris* may be due to lack of anthocyanin expression or function, which can activate the DFR and ANS genes in the flavonoid biosynthetic pathway, resulting in lowered cyanidin and pelargonidin content [34].

### 3.2. Key Genes in the Anthocyanin Pathway Affect Coloration in White and Pink Tea Flowers

Flavonols and anthocyanins are synthesized via the phenylpropanoid pathway, in which dihydroflavonol is turned into different metabolites via two different steps catalyzed by DFR or FLS. DFR can catalyze the conversion of dihydroquercetin to leucoanthocyanins, and is considered a crucial enzyme in the anthocyanin metabolic pathway [35]. FLS catalyzes dihydroflavonols into flavonols, which competes at a key branch point with DFR in the anthocyanin pathway [36]. Luo Ping et al. [37] proposed that disequilibrium in the expression of FLS and DFR genes modulated the accumulation of anthocyanins and flavonols in both white and red flowers of different species. Their deduction has already been verified using heterologous expression of the FLS genes in tobacco, in which transgenes inhibiting the expression of DFR promoted flavonol accumulation, resulting in white flowers. Many similar results have confirmed that DFR and FLS gene products compete for common substrates in the biosynthetic pathways of anthocyanins and flavonols, respectively, thereby determining red or white color formation of flowers [14,38,39].

Remarkably, DFR can efficiently catalyze the reduction of dihydrokaempferol (DHK) to leucopelargonidin, resulting in the accumulation of pelargonidin derivatives in flowers [40,41]. Furthermore, the presence and activity of F3′H in the flower can remove substrate for pelargonidin production. Inhibition activity of FLS gene can reduce enzymatic competition for DHK substrate, promoting pelargonidin accumulation in DFR transgenics [42].

Flavonoid-3′,5-hydroxylase (F3′5′H), a member of the cytochrome P450 family, is a central enzyme for the synthesis of 3′,5′-hydroxylated anthocyanins, compounds normally required for blue or purple coloration in flowers [43]. Zuo Li et al. [44] verified that metabolic flux converts DHK and dihydroquercetin (DHQ) to dihydromyricetin (DHM), committing the pathway to delphinidin biosynthesis in F3′5′H overexpression tobacco, shifting flower color from light pink to varying purple phenotypes. On the contrary, down-regulation of the F3′5′H gene leads to strong FLS activity and weak F3′H activity, yielding white rather than pink flowers in transgenic *Nierembergia sp*. [45]. These results suggested that there may exist substantial competition between colorless flavonols and colored anthocyanins, and that downregulation of FLS may be necessary to attain darker coloration in these flowers.

The results are consistent with previous observations in transgenic plants and ornamental plants [46,47,48]. Similarly, expression of F3′5′H in a petunia along with a petunia cytochrome b5 gene producing cyanidin derivatives in a carnation cultivar effectively resulted in increased production of delphinidin-based anthocyanins and subsequent color alteration in petals [49]. These result indicate that an enhanced electron transfer system to F3′5′H adequately out-competed the endogenous F3′H (and DFR) activities [30].

In the present study, two deferentially expressed DFR genes were identified during tea flower development; the expression level of DFRs in pink flower was 10-fold higher than that of white flower from the second stage to the fifth stage. Conversely, expression level of FLS was 5-fold higher on average in white flowers than in pink flowers from the third to the fifth stage. We posit that this disequilibrium of FLS and DFR expression resulted in the accumulation of anthocyanins in BTP pink tea flowers. Similar results have been found in development of *Camellia nitidissima* flower, where high expression level of FLS along with a very low expression level of DFR resulted in no anthocyanins detected in golden yellow *Camellia nitidissima* flower.

On the other hand, DFR can catalyze the reduction of DHK to leucopelargonidin, which leads to pelargonidin-derivative accumulation in flowers. This explains why BTP pink tea flower petals contained pelargonidin, however none was detected in ZJW white tea flowers. Moreover, the expression level of two F3′5′Hs in pink flowers was more than 3-fold higher than that of white flowers in the second and third stages, and the expression level of F3′H in pink flower was nearly 10-fold higher than that of white flower. The results indicate that the differential expression of F3′5′H and F3′H genes in pink and white flowers affected production of anthocyanins and subsequent formation of flower color. In addition, leucoanthocyanidin dioxygenase (LDOX), the downstream gene of DFR in the anthocyanin-biosynthesis pathway, encodes the enzyme ANS, which is responsible for catalyzing the conversion of colorless leucoanthocyanidins into colored anthocyanidins, a key step in anthocyanin and proanthocyanidin biosynthesis pathways [36,50]. The expression level of LDOX was more than 5-fold higher in pink flowers than in white flowers, which promoted the accumulation of colored anthocyanidins in BTP flowers. On the other hand, leucoanthocyanidin reductase (LAR) can convert leucocyanidin to (+)-catechin, and increased levels of insoluble proanthocyanidins [51]. The expression level of LAR was on average 5-fold higher in pink flowers than white flowers, explaining why proanthocyanidin A was detected in pink flowers, but not in white flowers.

### 3.3. Hub Genes Involved In Flavonoid Accumulation during Flower Development

Using WGNCA, a useful methodology for identifying the pairwise relationships among gene expression [52], we identified developmental stage-specific gene expression modules associated with flavonoids in tea flowers (Figure 5B). In the brown and purple module associated with flavonoids metabolites, nine structural genes involved in flavonoids biosynthesis pathway were identified as candidate hub genes. Several structural genes from the families of the hub genes (FLS, DFR, and FLR) are thought to be regulators of colored anthocyanins accumulation as previously described.

Interestingly, two GDP-L-galactose phosphorylase (GGP1 and GGP2) genes were previously unreported with regard to flower development and flavonoids accumulation in tea. Previous researchers have reported that GGP was an key enzyme in protecting plants against cold stress by maintaining ascorbate concentration and ascorbate redox state in higher plants [53,54]. In fact, functional and regulatory relationships between GGP and the flavonoid pathway have been discussed in Canadian sea buckthorn (*Hippophae rhamnoides L.).* The higher expression of GGP and flavonoid accumulation was observed during leaf and fruit development (RC-4 cultivars), indicating that GGP genes are indirectly involved in the synthesis and metabolism of flavonoid [55]. In the present study, the expression level of GGP1 gene was 10-fold in pink flower than in white flower. We believe that GGP may be playing a role in flavonoid accumulation during pink flower development.

Transcription factors have been thought to play critical roles in flavonoid biosynthesis by regulating structural gene expression. Particularly, photoreceptors (i.e., COP1), light-response effectors (i.e., HY5) and transcription factor complexes (MYB-bHLH-WD40) are believed to be involved in multi-level regulation of light-induced flavonoid biosynthesis [56].

Previous studies have confirmed that GATA transcription factor mediates the crosstalk between brassinosteroids and light-signaling pathways, indirectly relating to flavonoid biosynthesis [57,58]. The existence of light-regulatory-units in the promoter region of structural genes like FLS and F3′H suggest their direct regulation by GATA zinc finger transcription factor [59,60].

In our study, two light-regulatory genes were identified as major regulators of the core flavonoid accumulation genes in tea flowers, namely GATA transcription factor 4 (GATA4) and Ultraviolet-B receptor 8 (UVR8). Remarkably, the expression level of GATA4 was consistent with that of FLS in white and pink tea flower. In consequence, GATA4, as a specialized regulator of FLS, likely directly impacted expression of FLS, resulting in regulation of flower coloration. However, the results of this work can only reveal the integrative coordinated relationship in tea flower development. The molecular and cellular mechanisms of interactions between GATA4 and the promoter of FLS require further elucidation.

## 4. Materials and Methods

### 4.1. Plant Materials

The white-flowered Zijuan purple tea (*Camellia sinensis* var. *assamica*) and pink-flowered Baitang purple tea (*Camellia sinensis* L. var. *Baitang*) were grown in the tea plantation of Baitang town (Boluo county, Guangdong, China) under natural conditions. Each variety of fresh flower was collected from three trees on November 13, 2017. The flowers were divided into 5 different development stages according to their development status. Tea flower developmental stages included those of the mature flower bud (Stage 1, S1), pre-opening (Stage 2, S2), initial opening (Stage 3, S3), half bloom (Stage 4, S4) and full bloom (Stage 5, S5). Petal samples were separately gathered at the five distinctive opening stages mentioned above. Petal samples were then immediately frozen in liquid N2 and stored in a-80 °C refrigerator until further metabolome and transcriptome analysis. All petals were taken from ten floral buds and pooled to generate one biological replicate, and each sample included three biological replicates.

### 4.2. Extraction and Separation of Secondary Metabolites

#### 4.2.1. Total Anthocyanin Measurements

The total content of anthocyanin in tea petals was measured with a spectrophotometer (UV-160 Shimadzu, Japan), which is a modification of the method previously used by Kang Wei et al. [3]. In 10 mL of ethanol (containing 0.1 M hydrochloric acid) anthocyanins were extracted for 30 min at 60 °C with intermittent shaking. After centrifuging (FC-14C, Fastwin Bio-Tech Co. Ltd., Guangzhou, Guangdong, China) 3000 rpm for 5 min, the suspension was filtered and absorbance was measured at 650 620 and 530 nm. The anthocyanin content measurement was based on the formula: ∆A = (A530 − A620) − 0.1(A650 − A620). Total anthocyanin contents were calculated by the formula: total anthocyanin (µmol/g) = (∆A × 100)/(4.62 × sample weight). All data are presented as the mean ± SD (*n* = 3).

#### 4.2.2. Sample Extraction

Petal metabolites were extracted by 100mg powder samples in 70% aqueous methanol (1.0 mL) for 24 h at 4 °C. The extracts were centrifuged (10,000 g) for 10min at 4 °C, and then the supernatant was filtered with a microfiltration film before MS/MS analysis. The sample extracts (5 μL) were injected into an Ultra Performance Liquid Chromatography (UPLC, Shimadzu Co., Kyoto, Japan) system (Shim-pack UFLC SHIMADZU CBM30A system; MS, Applied Biosystems 6500 Q TRAP). Metabolite separation was performed on Waters ACQUITY UPLC HSS T3 C18 Column (1.8 µm, 2.1 mm × 100 mm) using mobile phase A (0.04% acetic acid in ultra-pure water) and mobile phase B (0.04% acetic acid in acetonitrile). Mobile phase A was decreased linearly from 95% at 0 min to 5% at 11 min, and then increased linearly from 5% at 12 min to 95% at 12.1 min, and then held at 95% until 15 min. The flow rate was maintained at 0.40 mL min^−1^, with column temperature maintained at 40 °C; injection volume: 2 µL. The effluent was alternatively connected to an ESI-triple quadrupole-linear ion trap (Q TRAP)-MS system. Linear ion trap (LIT) and triple quadrupole (QQQ) scans were acquired on an API 4500 Q TRAP LC/MS/MS system, equipped with an ESI Turbo Ion-Spray interface, operating in a positive ion mode and controlled by Analyst 1.6 software (AB Sciex). The operation parameters for the ESI source were as follows: ion source, turbo spray; source temperature, 500 °C, and ion spray voltage (IS) voltage, 5500 V. In addition, the ion source gas I (GSI), gas II (GSII), and curtain gas (CUR) were set at 55, 60, and 25.0 psi, respectively, and the collision gas (CAD) was set at high. Instrument tuning and mass calibration were performed with 10 and 100 µmol/L polypropylene glycol solutions in QQQ and LIT modes, respectively. QQQ scans were acquired during MRM experiments and collision gas (nitrogen) set to 5 psi. DP and CE for individual MRM transitions were done with further DP and CE optimization. A specific set of MRM transitions was monitored for each period according to the metabolites eluted within the period.

The UPLC and MS/MS analysis was performed by MetWare Biotechnology Co., Ltd. (Wuhan, China). Metabolite quantification was conducted using multiple reaction monitoring (MRM), as previously described by Dong et al. [61]. Metabolite identification was performed on the MWDB (Metware Database, Metware Biotechnology Co., Ltd, Wuhan, China) and metabolite information public database. Mass spectrometry data of the metabolite features were processed using the Analyst 1.6.1 software. A mixture sample was run for quality control after every ten samples to monitor the repeatability of the analysis process. The identified metabolites were subject to partial least squares (PLS) discriminant analysis. Metabolite compounds found to have significant dissimilarities in content were set with limits of variable importance in projection (VIP) ≥ 1 and fold change ≥ 2 or ≤ 0.5.

### 4.3. RNA Extraction and Transcriptome Sequencing

Total RNA was extracted from 1.0 g of petals of BTP and ZJW using Sangon Total RNA Purification Kit according the manufacturer’s protocol (Shanghai Sangon Biotechnology Co., Ltd., Shanghai, China). The RNA-seq sequencing and assembly were conducted by the Biomarker Technologies Corporation (Beijing, China). The library was constructed and sequenced on the Illumina HiSeq 2500 platform. Following the removal of low-quality sequence reads, clean reads were mapped to the reference genome sequence (http://www.plantkingdomgdb.com/tea_tree/) using HISAT2 program.

### 4.4. Gene Functional Annotation and Expression Level Analysis

In order to annotate the assembled unigenes, homology searches were conducted against seven databases, such as NR (NCBI non-redundant protein database), KEGG (Kyoto Encyclopedia of Genes and Genomes), COG (Clusters of Orthologous Groups of proteins), UniProtKB/Swiss-Prot (UniProt Knowledgebase), KOG database (EuKaryotic Orthologous Groups of proteins database), Pfam (Pfam protein families database), Ortholog database and GO (Gene Ontology) database. Levels of gene expression were estimated by FPKM (fragments per kilobase of transcript per million fragments mapped) method [62].

The DESeq R package (1.10.1) was used to examine the differential expression levels between the two flower groups at different developmental stages [63]. A fold change (FC) value ≥ 2 and a false discovery rate (FDR) < 0.01 were established for identifying differentially expressed genes (DEGs) [64]. The GOseq R package was used to perform Gene Ontology (GO) enrichment analysis of the differentially expressed genes (DEGs) based on hypergeometric distribution [65]. In order to identify enriched pathways of differentially expressed genes, the statistical enrichment of genes was tested using KOBAS in KEGG pathways [66]. The protein interaction of DEGs was predicted by BLAST, analyzing the DEGs sequences in the genome of a related species.

### 4.5. Identification of Hub Genes Using Weighted Gene Co-Expression Network Analysis

Weighted gene co-expression network analysis (WGCNA) R package software was used to identify hub genes involved in anthocyanin metabolism pathways. Altogether 35 flavonoids that were significantly correlated with flower color were selected for analysis. Modules whose distance is less than 0.25 were merged. Modules were created using the default settings, with the exceptions of min module size, power, and merge cut height being respectively set to 30, 14 and 0.25. Genes were then hierarchically clustered based on topological overlap similarity. Module Eigengenes (MEs) values were used to estimate correlation between modules and the selected trails. A module was selected when the correlation of genes and modules were more than 0.9. The networks were visualized using Cytoscape v3.6.1.

### 4.6. Quantitative Real Time PCR and Expression Validation

In order to validate the RNA Seq results, the quantitative real-time PCR (qRT-PCR) was performed on the petal samples at the five development stages as described by Rothenberg et al. [67]. Total RNA isolation was conducted using RNAqueous™ Total RNA Isolation Kit according to the protocol. Quantitative real-time PCR analysis was carried out using the LightCycler^®^ 480 II Real-Time System (LightCycler^®^ 480 II Roche cycler, Roche, Carlsbad, CA, USA) with a 96-well plate. The thermal profile for the PCR amplification was 95 °C for 5 min, followed by 40 cycles of 10 s at 95 °C and another 40 cycles at 60 °C for 30 s. The HieffTM qPCR SYBR Green Master Mix (No Rox) (Yaesen Biotech Co., Ltd., Shanghai, China) was used for all PCR reactions according to the instruction’s protocol. The qRT-PCR Primers were designed using the Primer Premier 5.0 software (Table S9). All qRT-RCRs analyses were conducted using three technical and three biological replicates, with the reference gene β-actin selected as the internal expression control. The expression level of different genes to the control was calculated according to the 2^−∆∆CT^ method.

### 4.7. Statistical Analysis

The correlation between qRT-PCR and RNA-seq was analyzed using SPSS software (V20.0). Some figures and tables were presented using Excel 2010. Transcriptome sequencing and metabolome data statistics and some tables and figures were prepared on biomarker cloud platform (Biomarker Biotechnology Co., Bingjing, China).

## 5. Conclusions

The objective of this study was to reveal the mechanism of anthocyanin accumulation and putative genes involved in the coloration in white and pink tea (*Camellia sinensis*) flowers. Three specific anthocyanin substances were identified, i.e., cyanidin O-syringic acid, petunidin 3-*O*-glucoside, and pelargonidin 3-*O*-beta-d-glucoside, which only accumulated in pink tea flowers, and could not be detected in white flowers. The RNA-seq results showed that high expression of DFR, as well as its downstream genes, ANS and LAR, during pink flower development contributed to anthocyanin accumulation, while the higher expression level of FLS during white flower development resulted in a significant reduction of anthocyanins. The results suggest that disequilibrium of FLS and DFR expression levels result in different levels of anthocyanin accumulation and coloration in white and pink tea flowers. Nine structural genes and several transcription factors were identified as hub genes involved in the anthocyanin pathway using WGCNA. The research of anthocyanin accumulation and coloration mechanisms would contribute to breeding pink-flowered tea cultivars.

## Figures and Tables

**Table 1:** Type and content of anthocyanins in white (ZJW) and pink (BTP) during tea flower development.

	CPS (Count Per Second)									
**Compounds**	**ZJW1**	**SE**	**ZJW2**	**SE**	**ZJW3**	**SE**	**ZJW4**	**SE**	**ZJW5**	**SE**
Cyanidin 3-*O*-glucoside	1.11 × 10^6 a^	6.56 × 10^5^	6.89 × 10^5 a^	7.57 × 10^5^	5.87 × 10^5 a^	6.09 × 10^5^	3.17 × 10^6 a^	5.38 × 10^6^	1.38 × 10^6 a^	9.29 × 10^5^
Peonidin	4.28 × 10^4 a^	8.24 × 10^3^	4.92 × 10^4 a^	5.69 × 10^3^	6.32 × 10^4 a^	2.12 × 10^4^	5.07 × 10^4 a^	1.85 × 10^4^	5.10 × 10^4 a^	3.56 × 10^3^
Cyanidin *O*-syringic acid	NA	-	NA	-	NA	-	NA	-	NA	-
Cyanidin 3-*O*-rutinoside	1.21 × 10^7 a^	2.60 × 10^6^	1.33 × 10^7 a^	3.79 × 10^5^	1.28 × 10^7 a^	2.65 × 10^5^	1.16 × 10^7 a^	1.26 × 10^6^	1.27 × 10^7 a^	8.96 × 10^5^
Cyanidin 3,5-*O*-diglucoside	2.08 × 10^7 a^	3.79 × 10^5^	1.81 × 10^7 a^	9.00 × 10^5^	1.84 × 10^7 a^	5.29 × 10^5^	1.68 × 10^7 a^	6.66 × 10^5^	1.72 × 10^7 a^	1.47 × 10^6^
Malvidin 3,5-diglucoside	1.23 × 10^4 a^	2.81 × 10^3^	1.26 × 10^4 a^	3.16 × 10^3^	1.24 × 10^4 a^	1.47 × 10^3^	1.01 × 10^4 a^	1.72 × 10^3^	1.10 × 10^4 a^	2.57 × 10^3^
Pelargonin	5.42 × 10^5 a^	1.31 × 10^4^	4.28 × 10^5 a^	5.54 × 10^4^	4.55 × 10^5 a^	5.85 × 10^4^	3.78 × 10^5 a^	5.16 × 10^4^	3.86 × 10^5 a^	2.30 × 10^4^
Petunidin 3-*O*-glucoside	NA	-	NA	-	NA	-	NA	-	NA	-
Pelargonidin 3-*O*-β-d-glucoside	NA	-	NA	-	NA	-	NA	-	NA	-
Cyanidin	1.04 × 10^7 a^	1.22 × 10^6^	1.20 × 10^7 a^	1.53 × 10^5^	1.28 × 10^7 a^	2.97 × 10^6^	1.20 × 10^7a^	8.50 × 10^5^	1.09 × 10^7 a^	5.03 × 10^5^
Procyanidin A2	NA	-	NA	-	NA	-	NA	-	NA	-
Procyanidin B2	5.50 × 10^7 a^	6.51 × 10^5^	5.45 × 10^7 a^	2.15 × 10^6^	5.42 × 10^7 a^	2.17 × 10^6^	5.00 × 10^7 a^	1.95 × 10^6^	5.25 × 10^7 a^	2.00 × 10^6^
Procyanidin B3	5.83 × 10^7 a^	6.43 × 10^5^	5.64 × 10^7 a^	2.61 × 10^6^	5.60 × 10^7 a^	2.15 × 10^6^	5.09 × 10^7 a^	9.29 × 10^5^	5.42 × 10^7 a^	2.55 × 10^6^
**Compounds**	**BTP1**	**SE**	**BTP2**	**SE**	**BTP3**	**SE**	**BTP4**	**SE**	**BTP5**	**SE**
Cyanidin 3-*O*-glucoside	7.04 × 10^7 b^	5.01 × 10^6^	5.54 × 10^7 b^	5.85 × 10^6^	5.55 × 10^7 b^	8.66 × 10^6^	4.33 × 10^7 b^	5.74 × 10^6^	5.48 × 10^7 b^	9.47 × 10^6^
Peonidin	2.43 × 10^4 a^	8.66 × 10^3^	1.74 × 10^4 a^	1.27 × 10^4^	1.64 × 10^4 a^	9.47 × 10^3^	1.46 × 10^4 a^	5.74 × 10^3^	2.37 × 10^4 a^	1.59 × 10^4^
Cyanidin *O*-syringic acid	1.60 × 10^7 a^	1.81 × 10^6^	1.29 × 10^7 a^	1.16 × 10^6^	1.37 × 10^7 a^	2.79 × 10^6^	1.05 × 10^7 a^	3.85 × 10^6^	1.53 × 10^7 a^	2.10 × 10^6^
Cyanidin 3-*O*-rutinoside	7.61 × 10^6 a^	4.59 × 10^6^	6.31 × 10^6 a^	3.53 × 10^6^	6.07 × 10^6 a^	3.59 × 10^6^	6.15 × 10^6 a^	2.23 × 10^6^	6.64 × 10^6 a^	1.39 × 10^6^
Cyanidin 3,5-*O*-diglucoside	1.54 × 10^7 a^	4.92 × 10^6^	1.21 × 10^7 a^	4.84 × 10^6^	1.16 × 10^7 a^	3.16 × 10^6^	1.03 × 10^7 a^	4.04 × 10^6^	9.06 × 10^6 a^	1.73 × 10^6^
Malvidin 3,5-diglucoside	2.62 × 10^4 a^	1.16 × 10^4^	2.21 × 10^4 a^	7.04 × 10^3^	2.36 × 10^4 a^	7.97 × 10^3^	1.86 × 10^4 a^	8.21 × 10^3^	2.06 × 10^4 a^	5.60 × 10^3^
Pelargonin	5.41 × 10^5 a^	2.99 × 10^5^	4.35 × 10^5 a^	1.62 × 10^5^	4.13 × 10^5 a^	7.31 × 10^4^	3.88 × 10^5 a^	2.06 × 10^5^	2.36 × 10^5 a^	5.64 × 10^4^
Petunidin 3-*O*-glucoside	5.46 × 10^7 a^	1.38 × 10^7^	2.55 × 10^7 a^	3.33 × 10^6^	2.23 × 10^7 a^	5.05 × 10^6^	1.41 × 10^7 a^	5.33 × 10^6^	1.51 × 10^7 a^	4.84 × 10^6^
Pelargonidin 3-*O*-β-d-glucoside	2.33 × 10^6 a^	1.10 × 10^6^	1.52 × 10^6 a^	5.74 × 10^5^	1.33 × 10^6 a^	2.41 × 10^5^	1.04 × 10^6 a^	5.08 × 10^5^	1.44 × 10^6 a^	3.79 × 10^5^
Cyanidin	1.28 × 10^7 a^	6.05 × 10^6^	1.32 × 10^7 a^	4.84 × 10^6^	1.38 × 10^7 a^	4.40 × 10^6^	1.38 × 10^7 a^	4.91 × 10^6^	1.21 × 10^7 a^	5.45 × 10^6^
Procyanidin A2	NA	-	8.02 × 10^4 a^	8.00 × 10^4^	1.61 × 10^5 a^	1.34 × 10^5^	2.10 × 10^5 a^	9.82 × 10^4^	1.51 × 105 ^a^	5.42 × 10^4^
Procyanidin B2	3.73 × 10^7 a^	8.26 × 10^6^	3.62 × 10^7 a^	6.37 × 10^6^	3.73 × 10^7 a^	8.65 × 10^6^	4.08 × 10^7 a^	3.07 × 10^6^	4.53 × 10^7 a^	3.53 × 10^6^
Procyanidin B3	3.98 × 10^7 a^	9.33 × 10^6^	3.87 × 10^7 a^	6.69 × 10^6^	3.86 × 10^7 a^	8.06 × 10^6^	4.21 × 10^7 a^	3.40 × 10^6^	4.54 × 10^7 a^	2.64 × 10^6^

Note: CPS, Count Per Second; SE, Standard Error; ZJW1, Zijuan white flower stage 1; ZJW2, Zijuan white flower stage 2; ZJW3, Zijuan white flower stage 3; ZJW4, Zijuan white flower stage 4; ZJW5, Zijuan white flower stage 5. BTP1, Baitang pink flower stage 1; BTP2, Baitang pink flower stage2; BTP3, Baitang pink flower stage 3; BTP4, Baitang pink flower stage 4; BTP5, Baitang pink flower stage 5. Each data is the mean of three biological replicates. Different letters of the same compound (i.e., ^a^ or ^b^) indicate significant differences (*p* < 0.05, by Duncan’s multiple range test).

## Supplementary Materials

Supplementary Materials are available online, Figure S1: DEMs statistics and Venn diagrams between white flower tea development stages, Table S1: Differentially expressed metabolites (DEMs) during white tea flower (ZJW) and pink tea flower (BTP), Table S2: Overview of the transcriptome sequencing dataset and quality check, Table S3: Clean reads mapped to the reference genome in tea flower development, Table S4: DEGs statistics in different cDNA libraries during pink tea flower development, Table S5: Primer sequences for qRT-PCR analysis, Table S6: Hub genes from the brown and purple modules, Table S7: KEGG pathway enrichment analysis of DEGs in pink tea flowers, Table S8: Highly expressed structural genes involved in the flavonoid pathway in white and pink tea flower development stages, Table S9: DEGs in brown and purple module, Table S10: KEGG pathway enrichment analysis of DEGs in white tea flowers.

## Data Accessibility

Raw sequencing data were deposited in the NCBI Bioproject database (accession number: PRJNA597123 and PRJNA597289).
