# Supplementary material for: Metabolome and Transcriptome Analysis Reveals Putative Genes Involved in Anthocyanin Accumulation and Coloration in White and Pink Tea (Camellia sinensis) Flower
Source: Molecules. 2020 Jan 2;25(1):190. doi: 10.3390/molecules25010190 (PMC6983220; doi:10.3390/molecules25010190)
Supplement: Supplementary file 1 [file molecules-25-00190-s001.zip › Supplementary S1 v2.pdf]

Supplementary S1

Table S1. Differentially expressed metabolites (DEMs) during white tea flower(ZJW) and pink tea flower(BTP)

	**group**	**DEMs_total**	**DEMs_up**	**DEMs_down**
Pvalue=0.05 FC=1 VIP=1	ZJW1_vs_ZJW2	260	135	125
	ZJW1_vs_ZJW3	245	132	113
	ZJW1_vs_ZJW4	338	173	165
	ZJW1_vs_ZJW5	350	200	150
	ZJW1_vs_BTP1	197	56	141
	ZJW2_vs_ZJW3	62	39	23
	ZJW2_vs_ZJW4	193	102	91
	ZJW2_vs_ZJW5	257	149	108
	ZJW2_vs_BTP2	223	72	151
	ZJW3_vs_ZJW4	79	47	32
	ZJW3_vs_ZJW5	155	96	59

	ZJW3_vs_BTP3	208	64	144
	ZJW4_vs_ZJW5	146	109	37
	ZJW4_vs_BTP4	188	63	125
	ZJW5_vs_BTP5	228	63	165
	BTP1_vs_BTP2	50	30	20
	BTP1_vs_BTP3	77	44	33
	BTP1_vs_BTP4	105	64	41
	BTP1_vs_BTP5	116	74	42
	BTP2_vs_BTP3	15	9	6
	BTP2_vs_BTP4	74	54	20
	BTP2_vs_BTP5	86	61	25
	BTP3_vs_BTP4	42	35	7
	BTP3_vs_BTP5	69	53	16
	BTP4_vs_BTP5	20	15	5

Figure S1

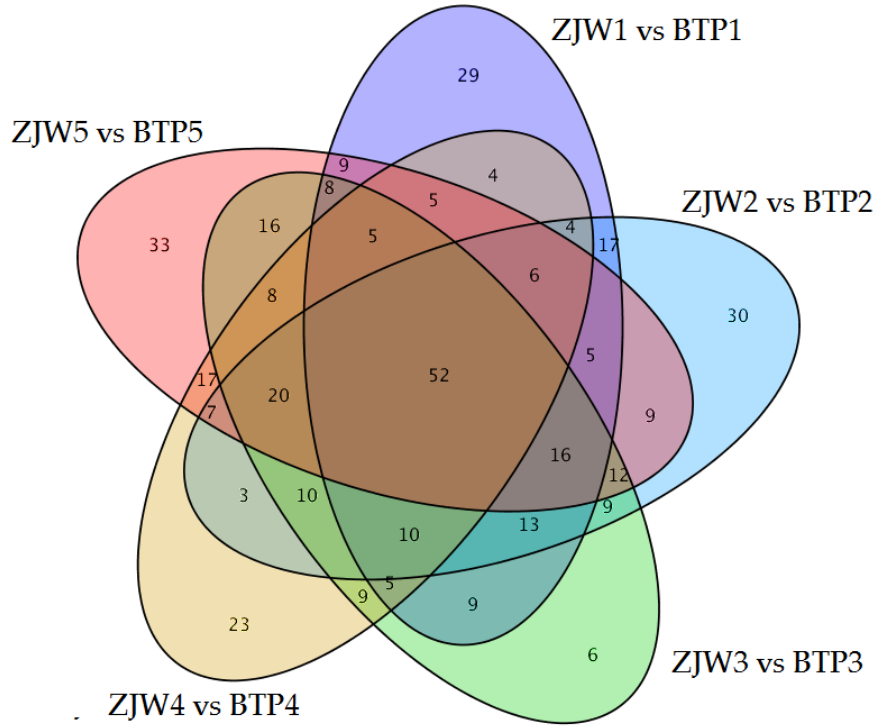

Figure S1. DEMs statistics and Venn diagrams between white flower tea development stages.

Table S2. Overview of the transcriptome sequencing dataset and quality check.

BMK-ID	Clean reads	Clean bases	GC Content	%≥Q30
BTP11	23,668,665	7,057,954,060	44.44%	86.78%
BTP12	26,153,281	7,804,636,930	44.36%	88.13%
BTP13	23,869,766	7,124,268,318	43.95%	87.54%
BTP21	25,455,270	7,579,737,926	44.29%	87.64%
BTP22	31,208,670	9,316,682,038	44.53%	89.02%
BTP23	23,867,889	7,096,074,108	44.76%	86.94%
BTP31	31,292,398	9,352,405,318	44.52%	88.85%
BTP32	26,972,499	8,016,173,786	44.56%	87.77%
BTP33	27,206,056	8,119,153,772	44.49%	88.16%
BTP41	30,166,304	8,954,093,392	44.55%	88.48%
BTP42	27,381,944	8,125,553,048	44.37%	88.07%

**BMK-ID**	**Clean reads**	**Clean bases**	**GC Content**	**%≥Q30**
BTP43	31,911,343	9,513,539,518	44.34%	88.67%
BTP51	21,561,262	6,436,558,604	45.34%	85.01%
BTP52	23,149,731	6,873,130,538	45.55%	87.02%
BTP53	23,033,043	6,867,835,118	44.93%	85.27%
ZJW11	24,401,173	7,272,491,122	44.91%	85.34%
ZJW12	21,739,300	6,470,253,566	44.38%	85.88%
ZJW13	21,682,289	6,448,733,710	44.57%	85.29%
ZJW21	20,915,131	6,229,932,294	45.22%	85.24%
ZJW22	22,420,616	6,674,212,962	44.78%	85.01%
ZJW23	21,883,981	6,513,593,344	44.73%	85.21%
ZJW31	20,935,452	6,220,494,474	45.17%	85.01%
ZJW32	21,362,041	6,362,838,760	45.30%	85.73%
ZJW33	23,929,435	7,138,488,744	44.90%	85.05%

BMK-ID	Clean reads	Clean bases	GC Content	%≥Q30
ZJW41	25,751,867	7,692,097,820	45.21%	87.27%
ZJW42	24,301,648	7,234,121,756	45.28%	88.20%
ZJW43	24,896,357	7,406,220,944	44.88%	88.16%
ZJW51	21,291,989	6,355,399,448	45.26%	87.70%
ZJW52	22,152,462	6,614,402,460	45.24%	87.49%
ZJW53	28,163,774	8,376,422,666	45.03%	88.19%

1–3: Three biological replicates of each tissue at tea flower development stage; Raw reads: original number of reads obtained by sequencing; Clean reads: number of reads after removing low-quality reads and trimming adapter sequences; Clean bases: number of clean reads multiplied by length of clean reads. Q30: Phred score, indicates 99% and 99.9% accuracy of sequenced bases; GC content: percentage of G and C in total bases.

Table S3. Clean reads mapped to the reference genome in tea flower development.

BMK-ID	Total Reads	Mapped Reads	Uniq Mapped Reads	Multiple Map Reads	Reads Map to '+'	Reads Map to '-'
BTP11	47,337,330	37,422,883 (79.06%)	36,178,993 (76.43%)	1,243,890 (2.63%)	18,577,511 (39.24%)	18,623,603 (39.34%)
BTP12	52,306,562	41,528,122 (79.39%)	40,141,768 (76.74%)	1,386,354 (2.65%)	20,608,596 (39.40%)	20,678,371 (39.53%)
BTP13	47,739,532	37,636,163 (78.84%)	36,399,620 (76.25%)	1,236,543 (2.59%)	18,686,335 (39.14%)	18,735,466 (39.25%)
BTP21	50,910,540	39,934,373 (78.44%)	38,579,973 (75.78%)	1,354,400 (2.66%)	19,797,265 (38.89%)	19,901,130 (39.09%)
BTP22	62,417,340	48,507,551 (77.71%)	46,952,204 (75.22%)	1,555,347 (2.49%)	24,058,387 (38.54%)	24,174,268 (38.73%)
BTP23	47,735,778	37,434,584 (78.42%)	36,167,403 (75.77%)	1,267,181 (2.65%)	18,552,881 (38.87%)	18,649,087 (39.07%)
BTP31	62,584,796	49,101,031 (78.46%)	47,554,132 (75.98%)	1,546,899 (2.47%)	24,377,166 (38.95%)	24,443,914 (39.06%)
BTP32	53,944,998	42,116,688 (78.07%)	40,744,631 (75.53%)	1,372,057 (2.54%)	20,886,414 (38.72%)	20,964,314 (38.86%)
BTP33	54,412,112	42,647,825 (78.38%)	41,334,061 (75.96%)	1,313,764 (2.41%)	21,161,580 (38.89%)	21,234,625 (39.03%)
BTP41	60,332,608	47,334,361 (78.46%)	45,735,146 (75.81%)	1,599,215 (2.65%)	23,472,221 (38.90%)	23,541,859 (39.02%)
BTP42	54,763,888	41,994,541 (76.68%)	40,595,379 (74.13%)	1,399,162 (2.55%)	20,823,492 (38.02%)	20,893,284 (38.15%)
BTP43	63,822,686	50,036,327 (78.40%)	48,382,053 (75.81%)	1,654,274 (2.59%)	24,823,383 (38.89%)	24,903,323 (39.02%)

BMK-ID	Total Reads	Mapped Reads	Uniq Mapped Reads	Multiple Map Reads	Reads Map to '+'	Reads Map to '-'
BTP51	43,122,524	31,208,842 (72.37%)	30,198,345 (70.03%)	1,010,497 (2.34%)	15,471,758 (35.88%)	15,548,724 (36.06%)
BTP52	46,299,462	34,863,566 (75.30%)	33,434,828 (72.21%)	1,428,738 (3.09%)	17,233,925 (37.22%)	17,344,370 (37.46%)
BTP53	46,066,086	34,243,367 (74.34%)	33,113,879 (71.88%)	1,129,488 (2.45%)	16,967,454 (36.83%)	17,052,198 (37.02%)
ZJW11	48,802,346	37,293,853 (76.42%)	36,008,165 (73.78%)	1,285,688 (2.63%)	18,493,535 (37.89%)	18,610,192 (38.13%)
ZJW12	43,478,600	32,962,390 (75.81%)	31,853,261 (73.26%)	1,109,129 (2.55%)	16,347,844 (37.60%)	16,433,698 (37.80%)
ZJW13	43,364,578	33,092,335 (76.31%)	32,013,598 (73.82%)	1,078,737 (2.49%)	16,402,973 (37.83%)	16,503,569 (38.06%)
ZJW21	41,830,262	31,589,683 (75.52%)	30,454,831 (72.81%)	1,134,852 (2.71%)	15,652,381 (37.42%)	15,767,875 (37.69%)
ZJW22	44,841,232	34,037,671 (75.91%)	32,892,099 (73.35%)	1,145,572 (2.55%)	16,870,426 (37.62%)	16,987,705 (37.88%)
ZJW23	43,767,962	33,392,416 (76.29%)	32,315,261 (73.83%)	1,077,155 (2.46%)	16,553,103 (37.82%)	16,670,821 (38.09%)
ZJW31	41,870,904	31,933,307 (76.27%)	30,830,111 (73.63%)	1,103,196 (2.63%)	15,834,315 (37.82%)	15,933,041 (38.05%)
ZJW32	42,724,082	32,989,600 (77.22%)	31,856,435 (74.56%)	1,133,165 (2.65%)	16,352,938 (38.28%)	16,465,577 (38.54%)
ZJW33	47,858,870	36,838,098 (76.97%)	35,628,007 (74.44%)	1,210,091 (2.53%)	18,256,902 (38.15%)	18,375,347 (38.39%)
ZJW41	51,503,734	39,406,493 (76.51%)	37,950,544 (73.69%)	1,455,949 (2.83%)	19,538,002 (37.94%)	19,622,512 (38.10%)

BMK-ID	Total Reads	Mapped Reads	Uniq Mapped Reads	Multiple Map Reads	Reads Map to '+'	Reads Map to '-'
ZJW42	48,603,296	36,630,157 (75.37%)	35,339,355 (72.71%)	1,290,802 (2.66%)	18,164,886 (37.37%)	18,245,513 (37.54%)
ZJW43	49,792,714	38,744,562 (77.81%)	37,392,013 (75.10%)	1,352,549 (2.72%)	19,211,077 (38.58%)	19,273,247 (38.71%)
ZJW51	42,583,978	32,400,843 (76.09%)	31,230,927 (73.34%)	1,169,916 (2.75%)	16,064,823 (37.73%)	16,136,608 (37.89%)
ZJW52	44,304,924	33,625,913 (75.90%)	32,446,166 (73.23%)	1,179,747 (2.66%)	16,688,854 (37.67%)	16,752,348 (37.81%)
ZJW53	56,327,548	43,193,996 (76.68%)	41,523,939 (73.72%)	1,670,057 (2.96%)	21,409,569 (38.01%)	21,504,543 (38.18%)

GC content: Clean Data GC content，the percentage of G and C base number in the total base number; ≥Q30: The percentage of base number which qualities of Clean Data is greater than or equal to 30 in the total base number, the number of Clean Reads is single ended calculation; Mapped Reads: The number of Reads which are compared to the reference genome and the percentage of this in Clean Reads; Uniq Mapped Reads: The number of Reads which are compared to the reference genome in single location and the percentage of this in Clean Reads.

Table S4. DEGs statistics in different cDNA libraries during pink tea flower development

#Parameters	group	DEGs_total	DEGs_up	DEGs_down
DESeq2_EBSeq FDR=0.01 FC=2	BTP11&BTP13&BTP12_vs_BTP21&BTP22&BTP23	5282	2138	3144
	BTP11&BTP13&BTP12_vs_BTP31&BTP32&BTP33	8945	4076	4869
	BTP11&BTP13&BTP12_vs_BTP41&BTP42&BTP43	12296	5286	7010
	BTP11&BTP13&BTP12_vs_BTP51&BTP52&BTP53	11557	4888	6669
	BTP11&BTP13&BTP12_vs_ZJW11&ZJW12&ZJW13	3254	1348	1906
	BTP21&BTP22&BTP23_vs_BTP31&BTP32&BTP33	2974	1987	987
	BTP21&BTP22&BTP23_vs_BTP41&BTP42&BTP43	8318	3786	4532
	BTP21&BTP22&BTP23_vs_BTP51&BTP52&BTP53	8306	3882	4424
	BTP21&BTP22&BTP23_vs_ZJW21&ZJW22&ZJW23	3827	1577	2250
	BTP31&BTP32&BTP33_vs_BTP41&BTP42&BTP43	3006	1241	1765
	BTP31&BTP32&BTP33_vs_BTP51&BTP52&BTP53	3795	1678	2117

#Parameters	group	DEGs_total	DEGs_up	DEGs_down
	BTP31&BTP32&BTP33_vs_ZJW31&ZJW32&ZJW33	5100	2046	3054
	BTP41&BTP42&BTP43_vs_BTP51&BTP52&BTP53	421	273	148
	BTP41&BTP42&BTP43_vs_ZJW41&ZJW42&ZJW43	3980	1870	2110
	BTP51&BTP52&BTP53_vs_ZJW51&ZJW52&ZJW53	3591	1587	2004
	ZJW11&ZJW12&ZJW13_vs_ZJW21&ZJW22&ZJW23	346	253	93
	ZJW11&ZJW12&ZJW13_vs_ZJW31&ZJW32&ZJW33	2275	1106	1169
	ZJW11&ZJW12&ZJW13_vs_ZJW41&ZJW42&ZJW43	6843	3287	3556
	ZJW11&ZJW12&ZJW13_vs_ZJW51&ZJW52&ZJW53	6331	2983	3348
	ZJW21&ZJW22&ZJW23_vs_ZJW31&ZJW32&ZJW33	44	33	11
	ZJW21&ZJW22&ZJW23_vs_ZJW41&ZJW42&ZJW43	4733	2450	2283
	ZJW21&ZJW22&ZJW23_vs_ZJW51&ZJW52&ZJW53	4768	2399	2369

#Parameters	group	DEGs_total	DEGs_up	DEGs_down
DESeq2_EBSeq FDR=0.01 FC=2	ZJW31&ZJW32&ZJW33_vs_ZJW41&ZJW42&ZJW43	2856	1411	1445
	ZJW31&ZJW32&ZJW33_vs_ZJW51&ZJW52&ZJW53	3206	1477	1729
	ZJW41&ZJW42&ZJW43_vs_ZJW51&ZJW52&ZJW53	23	14	9
	BTP11&BTP12&BTP13_vs_ZJW21&ZJW22&ZJW23	7860	3664	4196
	BTP21&BTP22&BTP23_vs_ZJW31&ZJW32&ZJW33	5740	2873	2867
	ZJW11&ZJW12&ZJW13_vs_BTP11&BTP12&BTP13	5060	2328	2732

Table S5 Primer sequences for qRT-PCR analysis

Gene	Accession	Forward Primer (5′to 3′)	Reverse Primer (5′to 3′)	product (bp)	Tm （℃）
LAR	CSNG5035	AGACGGACGGACAGACAAAG	GGAGTGGTGGAAGGACCGTA	176	60
LAR	CSA019984	ATGACTTACTTGCCGCTGCT	GGTAGGTTTGTCGAGGGCAT	116	60
FLS	CSA003707	CATGTACCCACCATGCCCAC	GAAGACCCGGAACGTCATTG	104	60
FLS	CSA006950	CGGGACTGGGGGATATTTCAG	TAGACCCAGGAGGCTTAGCA	136	60
F3'5'H	CSA031792	AGCGTGGGATGAAGAGGTTG	AGTGCCTTGATGTTGGTCGT	175	60
F3'H	CSA004930	CAATCACGTTCGCGGATATGT	ATTTCCAGCTTGGCCTTCAAA	130	60
DFR	CSNG45659	AGCGCCCTTTCTTTACAGTTTAT	AGGACAAGTAAAACACAAGTGGTAG	132	60
DFR	CSNG38209	TCGGTGTATTGGTGCCTTGC	GGCAAACTAAAACTCCGGTCA	170	60
DFR	CSA003949	CCAACCAAGTTTGAGAGTGTTGA	CTCCCACATCGTACTCATCCG	115	60
β-actin	KJ946252.1	TGCTGGCCGTGATCTAACAG	TGCTCGTAGTCAAGAGCGAC	135	60
